# Compression–Shear Specimen Stress-State Response and Distribution Characteristics with Wide Stress Triaxiality

**DOI:** 10.3390/ma17061424

**Published:** 2024-03-20

**Authors:** Yiwei Xu, Chunjiang Zhao, Chen Wang, Yunlong Qiu, Xiaosong Zhao, Shaolu Li, Ning Zhao

**Affiliations:** 1 School of Mechanical Engineering, Taiyuan University of Science and Technology, Taiyuan 030024, China; xuyiweiedu@163.com (Y.X.); wangc1215@163.com (C.W.); xs1464203901@163.com (X.Z.); 15536919488@163.com (S.L.); 15534249606@163.com (N.Z.); 2 Zhongxing Energy Equipment Co., Ltd., Nantong 226121, China; m18435151970@163.com

**Keywords:** compressive test, compressive shear compound loading, digital image correlation, ultra-low stress triaxiality

## Abstract

Conventional methods for studying the plastic behavior of materials involve uniaxial tension and uniaxial compression. However, in the metal rolling process, the deformation zone undergoes a complex loading of multidirectional compression and shear. Characterizing the corresponding plastic evolution process online poses challenges, and the existing specimen structures struggle to accurately replicate the deformation-induced loading characteristics. In this study, we aimed to design a compression–shear composite loading specimen that closely mimics the actual processing conditions. The goal was to investigate how the specimen structure influences the stress–strain response in the deformation zone. Using commercial finite element software, a compression–shear composite loading specimen was meticulously designed. Five 304 stainless steel specimens underwent uniaxial compressive loading, with variation angles between the preset notch angle (PNA) of the specimen and compression direction. We employed digital image correlation methods to capture the impact of the PNA on the strain field during compression. Additionally, we aimed to elucidate the plastic response resulting from the stress state of the specimen, particularly in relation to specimen fracture and microstructural evolution.

## 1. Introduction

During the plastic forming of metal parts, the deformation region undergoes a complex stress state, evident in processes such as extrusion [1], rolling [2], hydraulic expansion [3], and sheet metal stamping. This state is often characterized by multiple stress components, such as tension, compression, shear, and torsion. However, controlling local damage behavior during deformation becomes challenging due to the intricate loading conditions, directly impacting the processing costs and causing additional energy consumption.

The complexity of stress states in different processes makes it challenging to apply material models designed for one specific process to others. Astakhov [4,5] emphasized that even in cutting, a process primarily focused on fracturing materials, there is the involvement of deforming triaxial stress states to optimize the minimum energy required for cutting. Moreover, this process aims to minimize deformation energy to extend the life of cutting tools [6].

After reviewing the existing studies, it becomes apparent that most plastic deformation relies on stress and strain states, with fracture-limit thresholds determined by strain limits identified through stress triaxiality. A comprehensive summary of recent research in the plastic metal forming field reveals the existence of different damage and fracture mechanisms at both the macroscopic and microscopic dimensions [7]. This is particularly noticeable below the stress triaxiality cut-off value [8], a point that remains unclear. Notably, Khan and Liu [9] demonstrated the occurrence of ductile fracture under non-proportional biaxial compression in Al 2024-T351 that depended on stress triaxiality behavior. In the metal processing industry, common techniques involve rolling, forging, and pipe processing. Oblique rolling and spinning processes generate significant compressive hydrostatic stress, causing stress triaxiality to exceed the conventionally considered ductile damage stress triaxiality cut-off value [8]. In the lower stress triaxiality region, different ductile damage mechanisms exist; however, this area has been relatively underexplored, as noted by several scholars [10,11,12].

Stress triaxiality significantly influences various aspects of the plastic process. It can either promote or inhibit nucleation processes in phase transformation materials [13,14], impact the ability of transformation-induced plasticity (TRIP), influence work hardening due to the dynamic reversion of dislocations [15], affect the dislocation glide [16,17], and contribute to ductile damage fracture processes [9], among others. To generate multiple stress states, researchers have developed and validated various model structures. They have also utilized the specimen deformation-to-fracture method to confirm the impact of stress states on the complete plastic process and to establish fracture models. Hancock [18] pioneered the use of notched tensile specimens and conducting uniaxial tensile tests to obtain multi-axial stress states. Thomson [19] and Alves [20,21] employed notched drum and strip specimens to measure damage variables during the deformation process, varying stress triaxiality by altering the notch radius. Bao and Wierzbicki [22] enhanced upsetting, shear, and tensile experiments by incorporating a broader range of stress triaxiality. Driemeier [23] designed a biaxial stress loading specimen, whereas Kubík [24] used unidirectional notched compression specimens to obtain negative stress triaxiality, expanding the application of the uncoupled ductile fracture criterion. Liu [25] developed a new specimen design for studying the stress characteristics of low stress triaxiality, combining slatted specimens and shear-notched specimens. This design enables the measurement of stress in the tested area during tensile experiments.

In summary, compound stress loading stands out as a current research hotspot. Current specimen structures successfully accommodate both multiaxial and uniaxial loading, encompassing tension and shear. Nevertheless, in metal deformation processes like rolling and rotary forging, the deformation zone frequently undergoes a combination of multidirectional compression and compression–shear compound stress loading. It is noteworthy that this specific scenario has not been simulated using the aforementioned specimen structures up to the present date. Abushawashi [26] designed a planar compression shear specimen to obtain fracture trajectory, yet the design has the drawback of not considering the variation in stress-state distribution in the thickness direction and its impact on plasticity and damage.

Building on prior research, we have developed a plane-strain specimen featuring a preset notch angle. This design allows for the quantitative control of proportional compression and shear stress loading within the deformation zone. By adjusting the preset notch deflection angle of the specimen in alignment with the pressing down direction, various stress states can be achieved. However, the discrepancy in size between the compression plane and loading plane of the specimen structure results in a non-uniform stress distribution within the deformation zone. Consequently, further exploration is needed to understand the characteristics of non-uniform stress loading and distribution on the compression plane. This expansion is rooted in the examination of plane strain within the shear stress state (SSS), as conducted by Liu [25] and Abushawashi [26].

## 2. Materials and Methods

### 2.1. Experimental Design

**Figure 1:**
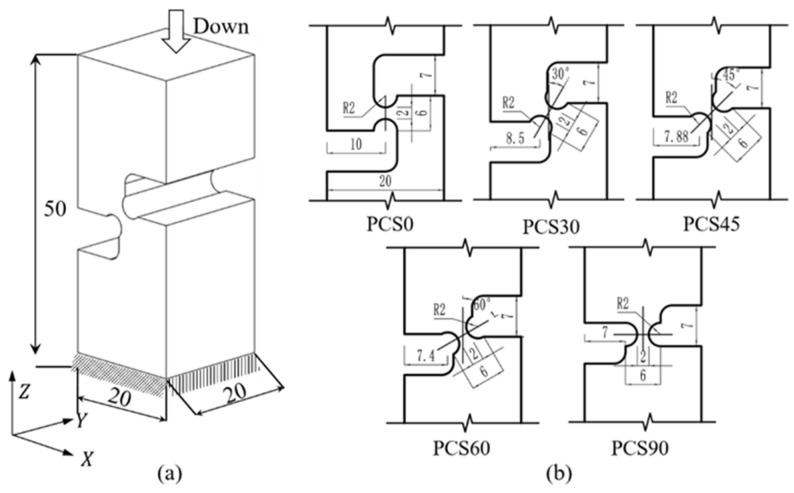
Geometric characteristics of the PCS specimens: (**a**) 3D structure of the PCS specimen and (**b**) geometry of specimens with varying notch inclinations.

To achieve the plane strain response characteristics in the compressive shear composite stress state, in this study, we conducted preset notched angle (PNA) compression experiments. The experiments utilize the plane-stress-state specimens enhanced by Liu [25] and Abushawashi [26]. The inclination structures of the specimens are obtained through horizontal, vertical, and multi-angle settings, covering medium, low, and very low stress triaxiality, as shown in Figure 1a. Subsequently, the digital image correlation (DIC) method is employed to acquire strain response results in the deformation region, ensuring the validation of the finite element method (FEM) characteristics.

To emphasize the stress state in the deformation zone, the reducer zone of the plane compression shear (PCS) specimen is intentionally designed to be much narrower than the specimen width. To create diverse stress triaxiality levels, the notch inclination is adjusted, resulting in different triaxial principal stress responses. This is accomplished by utilizing PCS specimens featuring two circular notches symmetrically placed at the center, each with inclination angles of 0°, 30°, 45°, 60°, and 90° concerning the specimen’s compression direction, as shown in Figure 1b. Ideally, the PCS0 deformation zone exhibits a pure shear, whereas PCS90 exhibits a uniaxial compressive stress state. The remaining specimens display various degrees of the compressive–shear compound stress state, which changes with the notch inclination.

**Figure 2:**
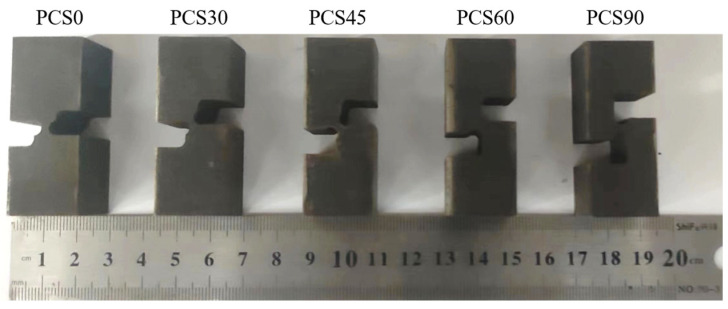
PCS specimen structure.

The material used for the experiment was 304 stainless steel of Taiyuan Iron & Steel (Group) Co., Ltd. (Taiyuan, Shanxi, China) with dimensions of 20 mm × 20 mm. The material composition is presented in Table 1. The specimen was initially heated to 1050 °C for 40 min and then subjected to high-temperature aging treatment at 820 °C for 8 h to obtain the specimen material. The specimen was processed using EDM wire cutting, as shown in Figure 2. The minimum position width of the reducer area is 2 mm, and its length is 4 mm. The fracture plane has an aspect ratio of 10 (=20 mm/2 mm), which is sufficient to generate a plane strain state.

The room temperature Johnson–Cook (J-C) model for 304 stainless steel, as described by Ling [27], was used as the elastic–plastic model in the FEM analysis. The model has undergone validation through compression and shear experiments, confirming its accuracy. Despite limitations in accurately capturing the plastic response to compression–shear compound loading, it can still be regarded as demonstrating a trend similar to the model. Ling’s model is shown as Equation (1):

(1) $$\sigma =\left[277+556\varepsilon^{0.794}\right]\left[1+0.0096\;\ln(\frac{\dot{\varepsilon }}{0.001})\right],$$

where σ represents the stress, ε represents the strain, and $\dot{\varepsilon }$ represents the ratio of strain.

This study is dedicated to investigating the stress response of PNA specimens under compression, with a specific focus on the compression plane. The study aims to offer insights that will facilitate the analysis of material microstructural evolution in subsequent research. However, it is crucial to note that the optimization of the J-C model, taking into account fracture damage, and the FEM simulation and validation processes are not covered in this paper.

### 2.2. Microscopic Mechanisms of Ductile Damage in Metallic Materials

After undergoing extensive plastic deformation, metal materials are generally prone to toughness fracture. This susceptibility arises from the combined effect of both the applied load and microstructure, which encompasses intergranular inclusions, second phase particles, and interfaces at various locations, such as matrix boundaries, phase boundaries, grain boundaries, and twin grain boundaries. These regions are particularly vulnerable to high-stress concentration states, often caused by dislocation plugging. This, in turn, triggers the nucleation of cavities and the formation of microcracks. Consequently, the material’s load-bearing capacity is compromised and microcracks elongate in the direction of deformation, eventually leading to material separation and, ultimately, fracture.

**Figure 3:**
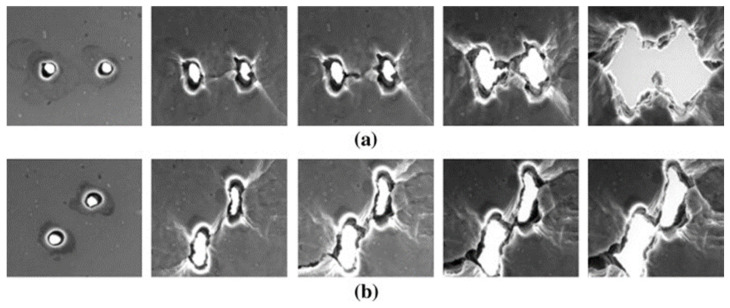
Two kinds of mechanisms for the coalescence of voids: (**a**) necking of inter-void ligaments and (**b**) shear-linking of voids (according to Weck and Wilkinson) [31].

Macroscopically, the fracture of metallic materials is influenced by various factors, such as the loading path, stress state, and material anisotropy. Among these factors, the stress state directly impacts the damage characteristics and mechanisms [28], as illustrated in Figure 3. High positive stress triaxiality conditions during tensile loading result in inter-pore necking and microscopic damage characterized by microcavity nucleation, growth, and consolidation, primarily due to the maximum principal stress. Conversely, under pure shear and compressive loading conditions featuring positive or negative stresses in triaxial dimensions, microporosity becomes connected through shear, extending in the direction of maximum shear stress [29]. Noell [30] hypothesized that the fracture toughness process is governed by multiple mechanisms, which may differ based on the material and stress conditions.

### 2.3. Stress-State Representation

#### 2.3.1. Method of Determining the Stress State of Metal Materials

Referring to Section 2.2, it is demonstrated that the stress state is one of the primary factors that affects the plasticity behavior of metallic materials. Various studies have consistently shown that shear fracture damage prevails, particularly in scenarios characterized by low stress triaxiality. The stress triaxiality, denoted by η (as outlined in Equation (2)), and the Lode angle parameter [25,31], denoted by $\bar{\theta }$ (as presented in Equation (3)), serve as standard parameters to depict the impact of planar compression loading on pore nucleation, growth, and aggregation under conditions of low stress triaxiality. The functional relationship is expressed as follows:

(2) $$\eta =\frac{I_{1}}{\left(3\sigma_{e}\right)},$$

(3) $$\bar{\theta }=1-6\theta /\pi =1-2arccos\xi \left(\theta \right)/\pi ,$$

where $I_{1}$ represents the third invariant of the spherical stress tensor, $\sigma_{e}$ denotes the equivalent effect stress, θ stands for the Lode angle, and ξ(θ) normalizes the dimensionless numbers, related to the Lode angle θ and the third invariant of the partial stress tensor $J_{3}$, which expressed in the form Equation (4):

(4) $$\xi \left(\theta \right)=cos\left(3\theta \right)=27J_{3}/2\sigma_{e}^{3},$$

Equations (2)–(4) are expressed in the principal stress space and the three stress tensor invariants $I_{1}$ and the partial stress tensor invariants $J_{2}$ and $J_{3}$ or dimensionless parameters between them, are described as:

(5) $$I_{1}=\sigma_{1}+\sigma_{2}+\sigma_{3}=3\sigma_{m},$$

(6) $$J_{2}=\frac{1}{6}\left[{\left(\sigma_{1}-\sigma_{2}\right)}^{2}+{\left(\sigma_{2}-\sigma_{3}\right)}^{2}+{\left(\sigma_{3}-\sigma_{1}\right)}^{2}\right]=\sigma_{e}^{3}/3,$$

(7) $$J_{3}=\left(\sigma_{1}-\sigma_{m}\right)\left(\sigma_{2}-\sigma_{m}\right)\left(\sigma_{3}-\sigma_{m}\right),$$

where $\sigma_{m}$ represents the hydrostatic pressure.

**Figure 4:**
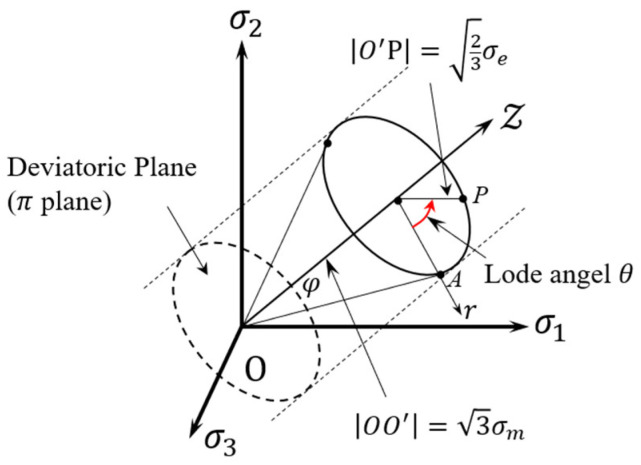
Geometric definition of the Lode angle θ in the principal stress space.

The equation takes values within the range of 0 to 1, whereas the Lode angle ranges from 0 to π/3, show as red arrow in Figure 4. The significance of the Lode angle θ is illustrated in Figure 4. In Section 2.3.2, we delve into the determination of numerical characteristics for a multi-directional loading stress state and a multi-axial stress state based on the Lode number distinction between simple stress states.

To describe the stress state, three coordinate systems are employed, with $\sigma_{1}$, $\sigma_{2}$, and $\sigma_{3}$ representing the triaxial principal stresses, commonly recognized as $\sigma_{1}\;$ ≥ ${\;\sigma }_{2\;}$ ≥ $\;\sigma_{3}$. These systems are the right-angle coordinate system with stress components ($\sigma_{1}$, $\sigma_{2}$, $\sigma_{3}$), the column coordinate system with components $\left(\sigma_{m},\;\sigma_{e},\theta \right)$, and the spherical coordinate system $\left(\sigma_{e},\eta ,\theta \right)$. Utilizing Figure 4, the stress triaxiality can be expressed as a function of *φ* [32], as shown in Equation (8):

(8) $$\eta =(\sqrt{2}cot\varphi )/3,$$

#### 2.3.2. Multidirectional Stress Loading Evaluation Method

**Figure 5:**
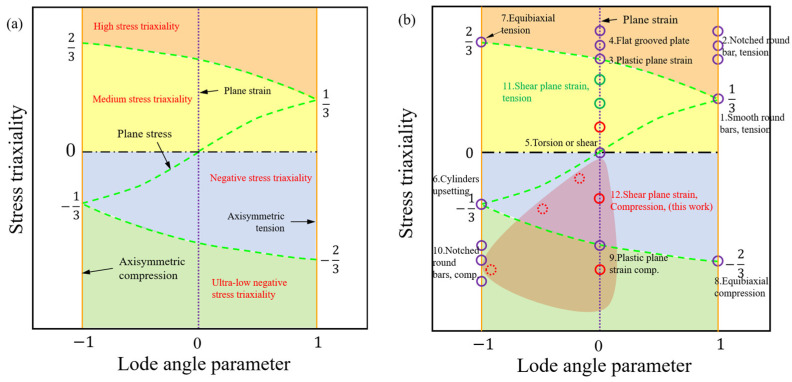
Specimen types and their corresponding stress states, plotted on the plane of stress triaxiality versus Lode angle parameter: (**a**) schematic of stress state definition and (**b**) schematic of the correspondence between the specimen and the stress state.

The parameter $\bar{\theta }$ takes a range of values $-1<\bar{\theta }<1$. The strain response for all loading conditions can be defined in the stress triaxiality and Lode angle parameter space $\left(\eta ,\;\bar{\theta }\right)$. Classical specimens describing plastic and damage processes under multiple stress states can be represented in the parameter space $\left(\eta ,\;\bar{\theta }\right)$, as shown in Figure 5 and Table 2. The stress state region covered of this study is shown as the red zone in Figure 5. Specifically, when θ=1, this corresponds to axisymmetric tension, θ=0 corresponds to generalized shear loading (planar plastic strain), and θ=−1 corresponds to axisymmetric compression. Notably, in planar stress states, Xue and Wierzbicki [33] proved that the condition $\sigma_{3}=0$ enables the parameters η and $\bar{\theta }$ (or ξ(θ)) as follows:

(9) $$\xi \left(\theta \right)=cos\left[\pi \left(1-\bar{\theta }\right)/2\right]=-27\eta \left(\eta^{2}-1/3\right)/2,$$

The position of the specimen in $\left(\eta ,\;\bar{\theta }\right)$ space, as shown in Figure 5, is denoted by a hollow circle representing three roots of the function. These roots correspond to the pure SSS $\left(\eta =0,\bar{\theta }=0\right)$ and the transverse plastic plane strain $\left(\eta =\pm 1/\sqrt{3},\;\bar{\theta }=0\right)$, effectively dividing the $\left(\eta ,\;\bar{\theta }\right)$ space into three regions. In Figure 5a, it is evident that the region where $\eta >1/\sqrt{3}$ is commonly referred to as the high-stress region. The area within $1/\sqrt{3}>\eta >0$ is termed the medium stress triaxiality region, encompassing uniaxial tension, shear, and torsional deformation. Two primary stress states emerge: the first, located at $-1/\sqrt{3}<\eta <0$, is identified as the low stress triaxiality region and includes uniaxial compression, isobiaxial compression, and compression–shear complex stress states. The second, situated at $-1/\sqrt{3}>\eta$, constitutes the ultra-low stress triaxiality region, where the main fracture mechanism is the hole-type collapse of the notched compression shear specimen. These distinctions are based on the representation of the plane stress loading state, depicted by the green dashed line in Figure 5. Table 1 provides a quantitative description of the specimen’s structure, characterizing multiple stress states with stress triaxiality η and Lode angle parameter $\bar{\theta }$, and is visually represented in the $\left(\eta ,\;\bar{\theta }\right)$ space in Figure 5b.

## 3. Planar Compression FEM Simulation and Experiment

### 3.1. Experimental Procedure

**Figure 6:**
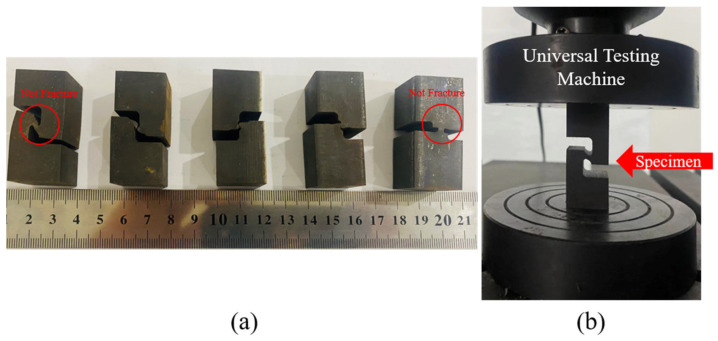
Plane compressive shear (PCS) specimen: (**a**) specimens were compressed to its limit position and (**b**) position of the specimen during compression.

The intricate geometry of PCS specimens poses challenges in directly assessing the stress state during deformation. Consequently, it becomes imperative to determine the stress distribution within the plastic deformation of the specimen and assess the stress state through the application of the FEM. Utilizing the model depicted in Figure 1b, compression was applied in one direction at a rate of 0.01 mm/s through Abaqus 6.14/Explicit commercial FEM software and a 30-ton hydraulic universal testing machine. This procedure aimed to generate compression specimens with deformations ranging from 1 mm to 1.5 mm, extending up to the point of fracture or the deformation limit positions, as illustrated in Figure 6. The primary objective of this experiment was to validate the plasticity and damage process of multiple PCS specimens. Considering the substantial difference in size between the deformation zone and the specimen substrate, it is postulated that the displacement–load data recorded by the built-in sensor of the universal testing machine accurately represents the displacement–load data of the deformation zone.

To validate the results obtained through FEM plasticity analysis, the DIC method was employed. This approach aimed to capture the strain field distribution outside the XZ section of the PCS specimen. The objective is to compare this captured distribution with the results obtained from FEM, aiming to validate the accuracy of the FEM model. Originally proposed by Peters [34], the DIC method has found extensive application in experimental strain studies. The equipment model utilized for the DIC method is VIC-3D, and detailed studies are presented in Section 3.2. The experimental findings reveal that the PCS30, PCS45, and PCS60 specimens fractured under stress, whereas the PCS0 and PCS90 specimens were compressed to their limits without breaking. Comprehensive accounts of the compression experiment results and data analysis are described in Section 4.

### 3.2. Experimental Results

**Figure 7:**
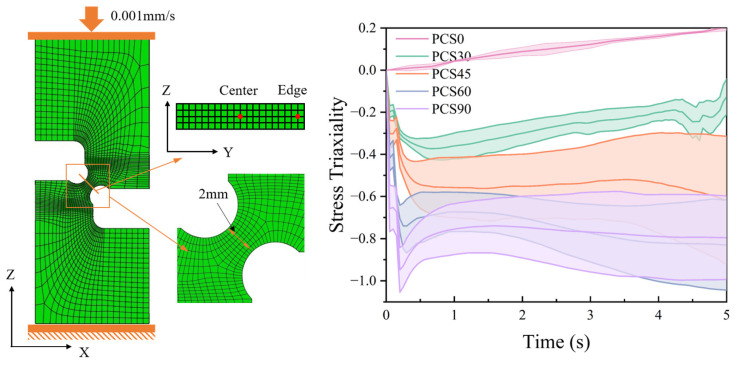
Evolution of stress triaxiality during compression of the PCS specimens.

This study delves into the investigation of the plastic process and stress response characteristics under planar compressive shear deformation. According to Section 1, the study of stress triaxiality ductile damage in metallic materials involves various parameters, such as the determination method for the stress-state evaluation factor in the stress triaxiality and Lode angle parameter of the deformation zone. The specific methodology involves the extraction of tri-axial principal stress within the deformation zone using Equations (5) and (6) to ascertain the stress-state characteristics, as depicted in Figure 7. This illustration reveals the stress-state distribution across the width of the deformation zone during compression, showcasing lower stress in the middle and relatively high stress at the edges.

#### 3.2.1. FEM Result

The FEM was executed at a loading rate of 0.1 mm/s. The FEM adopts the J-C model and parameters suitable for uniaxial compression and shear (refer to Equation (1)). The contact boundary conditions of the specimen are shown in Figure 6, where the surface friction coefficient is set to 0.15 [35] (indicating non-lubricated steel contact). The stress response in the Y-direction within the deformation zone of the PCS specimen is considered. Figure 7 displays the distribution of stress triaxiality and Lode angular parameters extracted from the center to the edge position in the neutral plane position of the YZ section within the PNA region. As for the compression deflection angle, the stress triaxiality undergoes a transition from a state of medium stress triaxiality to a state of ultra-low stress triaxiality. This shift is marked by a conspicuous delamination structure, underscoring the deflection angle’s significant impact on stress triaxiality. Throughout the deformation process, the stress state exhibits complex characteristics. Initially, it aligns with the predetermined stress triaxiality value of the structure. As deformation progresses, the PNA adjusts towards the compression direction, leading to a gradual increase in the notched deflection stress triaxiality.

The regional map depicting the distribution of transverse stress triaxiality within the deformation area, as shown in Figure 7, indicates similar deformation trends at both the edge and center positions. However, the distinction lies in the magnitude of the values.

**Figure 8:**
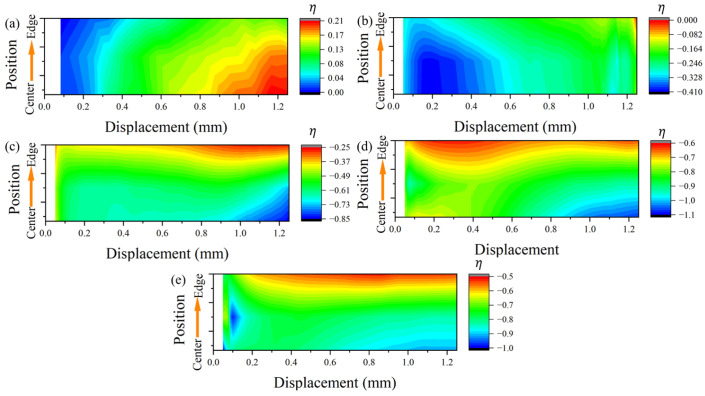
Evolutionary characteristics of the transverse stress triaxiality with deformation in the deformation zone: (**a**) PCS0, (**b**) PCS30, (**c**) PCS45, (**d**) PCS60, and (**e**) PCS90 (printed in color).

**Figure 9:**
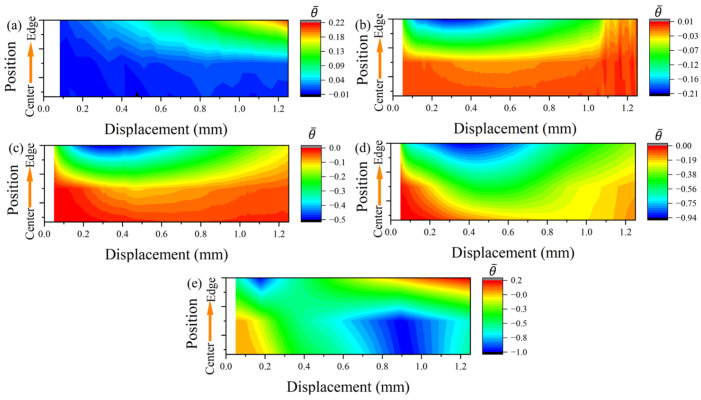
Evolutionary characteristics of the transverse Lode angle parameters with deformation in the deformation zone: (**a**) PCS0, (**b**) PCS30, (**c**) PCS45, (**d**) PCS60, and (**e**) PCS90 (printed in color).

Compared to the plate-shear specimens [36], the plane-strain specimens exhibit a significant difference in stress state in the transverse deformation direction [26]. The stress loading path and strain field crack heavily influence the path of crack extension. In the case of PCS specimens, their symmetric structures are often much larger than their thickness dimension in the width direction during deformation. To comprehensively understand the stress state, we carefully analyzed the semi-profile structure in the deformation zone. The evolution of the stress triaxiality (Figure 8) and the Lode angle parameter (Figure 9), with deformation in the width direction spanning from the center to the edge position of the deformation zone, was meticulously examined.

Throughout the compression process in the universal testing machine, the specimens undergo continuous plastic deformation. This deformation was characterized by changes in the distribution of stress-state parameters within its loading process as the compression amount increases. The planar pure shear specimen corresponding to PCS0 initially experiences the SSS of planar strain deformation at a displacement of 0.2. Subsequently, due to the increasing deflection angle, the plastic process unfolds with tensile stress-state characteristics, as depicted in Figure 8a. In the later stages of deformation, notable disparities in stress states emerge between the central and edge locations. While both plane strain tensile and SSS are present, the axisymmetric tensile effect is more pronounced at the edge location.

The same phenomenon is observed in the PCS30 specimen but with the distinction that the transverse direction of the deformation zone undergoes plane compressive strain. In comparison to the PCS0 specimen, the presence of an initial PNA subjected the deformation zone to compressive stress during the Y-direction loading process. Additionally, the compressive effect of the deformation zone becomes more pronounced with the increasing PNA.

The PCS specimens undergoing compressive loading exhibit a distinctive loading characteristic characterized by strong axisymmetric compressive strain at the edge position, especially before reaching a strain of one. A noticeable contrast in stress triaxiality and SSS is observed between the PCS45 specimens and PCS60 specimens, as illustrated in Figure 8c,d. The non-axisymmetric compressive deformation occurs from the central position, featuring compressive shear plane strain, and progresses towards the edge position, where an SSS prevails. The compression effect at the edge region becomes more pronounced with an increase in the initial specimen PNA (refer to Figure 9b–d). However, as the compression state weakens, the stress triaxiality at the edge surpasses that at the central region.

This observation leads to the conclusion that the ultra-low stress triaxiality is influenced by both the deformation characteristics and the SSS during planar deformation. In contrast, the PCS90 specimens, resembling approximately uniaxially compressed specimens, exhibit planar strain characteristics in the transverse direction, unlike conventional cylindrical specimens [37]. The central position of the PCS90 specimen consistently maintains a state of ultra-low stress triaxiality during compression. The biaxial compression strain characteristics are dominated by the axisymmetric compressive strain. Initially, the edge position experiences axisymmetric compressive strain and transitions to a pure SSS during the middle and later stages of deformation.

As the PNA gradually transitions from a planar shear state with 0° declination in the depression direction to a planar compression stress state with 90° declination in the depression direction, the stress triaxiality (η) at the center of the deformation zone decreases from 0.2 to −0.8. The detailed stress states in the deformation zones of each specimen are meticulously presented in Table 3, providing information on the average stress triaxiality in both the center and edge regions, along with the Lode angle parameter ($\bar{\theta }$).

#### 3.2.2. DIC Results

**Figure 10:**
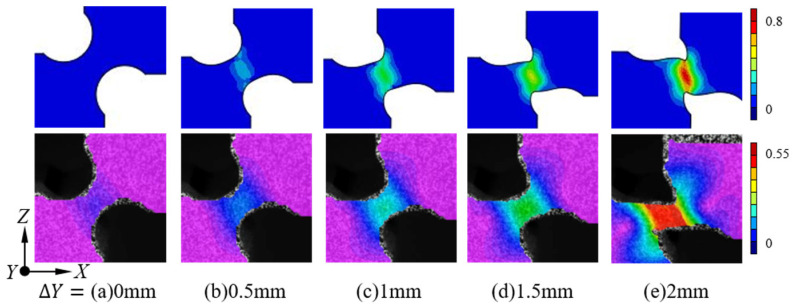
Comparison of FEM results and DIC results for the strain field during compression of the PCS45 specimens (**a**–**e**) (printed in color).

A comparison of the FEM result and the change in the hole pattern at the edge of the deformation zone during different compression strokes of the sample compression process is presented in Figure 10. Analyzing the strain field distribution characteristics in the deformation zone reveals concentration at the minimum section width location. The equivalent plastic strain emerges as a crucial parameter for characterizing the damage factor. The DIC strain field results indicate that the FEM responds to the actual compression deformation process with similar strain trends under the same compression amount. However, there are numerical differences between the two. Therefore, we can conclude that the employed FEM model effectively captures the actual deformation trends exhibited by the PCS specimen throughout the compression process, validating the accuracy of its FEM results. From the figure, it is evident that the equivalent strain gradually accumulates with increasing deformation along the Z-axis, consistently reaching its maximum at the center of the thickness direction. This position serves as the initiation point for microcrack sprouting and expansion [26].

Due to disparities in the friction coefficients set in the FEM and the actual friction coefficients between the contact surfaces, discrepancies emerge between the DIC results depicting the specimen’s deformation and the corresponding FEM results. To assess the accuracy of the FEM results, we extracted the DIC strain ($\varepsilon_{exm}$) and FEM strain ($\varepsilon_{FEM}$) in the XZ plane strain field at the same displacement. The relative percentile error (RPE) between the two is then calculated as follows:

(10) $$RPE=\left(\varepsilon_{FEM}-\varepsilon_{exm}\right)/\varepsilon_{exm},$$

Subsequently, the root mean squared relative percentile error (RMSRPE) for the entire set of data is calculated as follows to evaluate the stress predicting accuracy of the FEM model:

(11) $$RMSRPE=\sqrt{\sum_{i=1}^{n}{RPE}_{i}^{2}/n},$$

where n is the number of tests in the group.

As shown in Table 4, for this set of tests the maximum RPE is 12.9% and the RMSRPE is 7.72%, with the comparative results of DIC and FEM maximum strain for PCS specimens at a tester displacement of 1 mm. All RPE values are positive. However, we observe an increasing intensification of compressive loading in the deformation zone with the rise in PNA. This trend leads to a gradual increase in RPE, thereby diminishing the accuracy of the FEM results. However, the RMSRPE is less than 10% and, overall, the FEM has a better prediction accuracy for the stress response.

**Figure 11:**
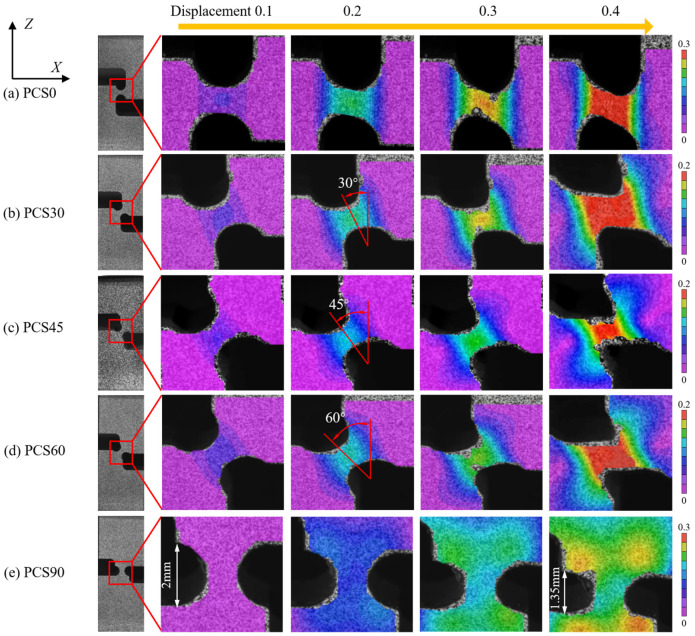
DIC observation of the relationship between the notched compression process and the strain field of PCS specimens (**a**–**e**) (printed in color).

As deformation progresses, the force characteristics of the deformation zone shift from a planar shear state to a planar tensile state, a transition supported by the strain distribution during the deformation process of the PCS0 specimen, as shown in Figure 11a. The DIC data undergo processing using the open-source NCORR image processing program, calculating the strain of the grid cells and providing deformation results, as shown in Figure 11. The color bar in the figure indicates the change in the value of the strain field in the DIC results.

Figure 11a–d depicts the four stages of the strain field in the various regions during successive loading. Using the equivalent strain of 0.2% as the cut-off point for material yielding, the first stage transpires before reaching this threshold. In this initial stage, deformation originates in the central region at the position of minimum thickness and subsequently propagates to the peripheral region. The second stage marks the material entering the yielding phase, with the accumulated strain increasing linearly with the PNA. As the third stage commences, the material transitions to the post-yielding phase. The PNA deflects in the Y-direction under compression, and the maximum strain direction rotates in alignment with the PNA. Despite this rotation weakening the compressive effect, the material still undergoes a robust compressive load due to the influence of the PNA. In the fourth stage, the PCS0 specimen (Figure 11a) undergoes tenuous deformation in the Y-axis loading direction, extending the fracture strain limit.

For the PCS30, PCS45, and PCS60 specimens (Figure 11b–d), different shear strain components in the principal strain and Y-direction angle are observed due to variations in the PNA, continuing until fracture occurs. The PCS90 specimen resembles the notched compression specimen [24], as both involve a deformation zone subjected to multidirectional compressive loading. The loading path for the specimen follows uniaxial compression along the Y-direction.

**Figure 12:**
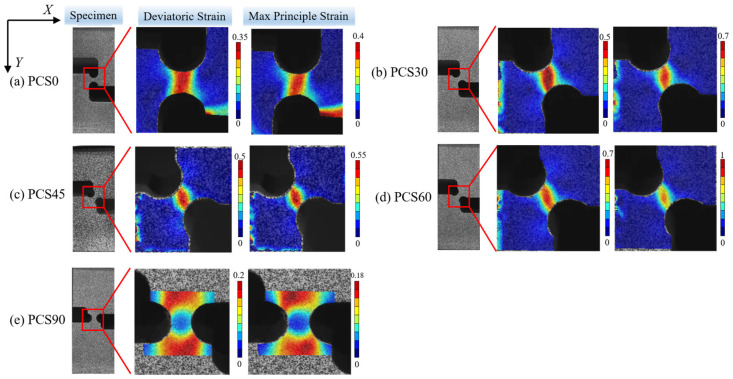
Strain field distribution of deviatoric strain and principal strain in PCS specimens: (**a**) PCS0, (**b**) PCS30, (**c**) PCS45, (**d**) PCS60, and (**e**) PCS90 (printed in color).

The NCORR open-source program was used to analyze the characteristics of the deviatoric strain ($\gamma_{xy}$) and the first principal strain ($\varepsilon_{1}$) in the deformation zone of PCS specimens, shown as Figure 12. The types of strain in the XY section of the specimen encompass shear strain, and compressive strains are present along the Y-axis and X-axis. We assume that no strain was generated along the X-axis due to friction force on the contact surface. The analysis reveals a clear increase in $\gamma_{xy}$ from 0.35 for the PCS0 specimen to 0.7 for the PCS60 specimen, with the principal strain rising from 0.4 to 1. The ratio of $\gamma_{xy}$ to $\varepsilon_{1}$, used to express the degree of shear deformation, decreases from 0.875 to 0.7. It can be inferred that shear deformation was suppressed with the increase in PNA, whereas compressive deformation increases more rapidly. The PCS90 specimen resembles a uniaxial compression specimen with no deviatoric strain generated in the deformation zone. This observation aligns with the anticipated outcomes of the specimen structure design.

#### 3.2.3. Compression Result

**Figure 13:**
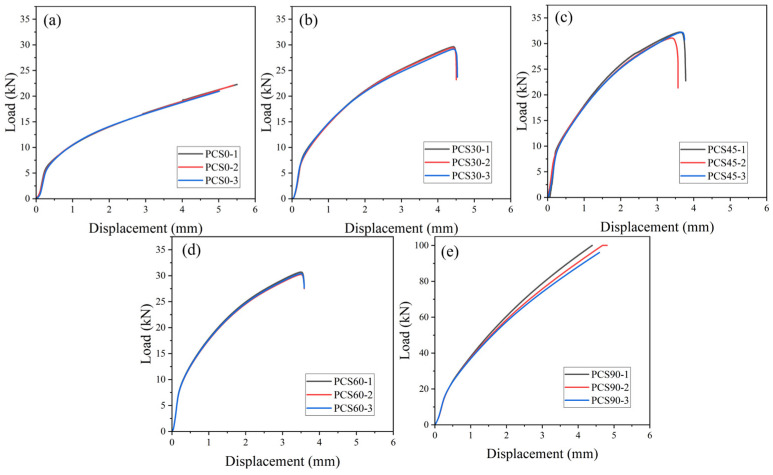
Extraction of the load–displacement plot of the testing machine: (**a**) PCS0; (**b**) PCS30; (**c**) PCS45; (**d**) PCS60; and (**e**) PCS90.

Compression experiments were conducted on notched specimens using a 30-ton hydraulic universal testing machine (refer to Figure 6b). The original specimen used in the experiment is shown in Figure 1a. The results for each compressed specimen are shown in Figure 6a (the load–displacement curve is shown in Figure 13), and the characteristic parameters for each specimen are detailed in Table 5. There are significant differences in loading force stratification, resulting in deformation zones with different compressive loading and offset load characteristics. With an increase in the PNA, the fracture displacement decreases, whereas the fracture load increases. It is important to mention that the PCS0 and PCS90 specimens experience planar tensile and uniaxial compressive stress, respectively, during the deformation process in the above analysis. Consequently, no fracture occurs under the limited compressive deformation stroke, explaining the absence of a fracture inflection point for the PCS0 and PCS90 specimens in Figure 13a,e.

The multi-PNA specimens utilized in this paper focus on studying the stress state and fracture behavior of 304 stainless steel under PCS deformation. While valuable for this purpose, these specimens have limitations in providing a comprehensive depiction of the fracture damage behavior of 304 stainless steel under various stress states. To address these limitations, we consulted several studies conducted by researchers on 304 stainless steel that utilized various structural specimens to investigate damage strain under different loading and deformation characteristics, such as uniaxial tensile, uniaxial compressive, multi-axial tensile, torsion, shear, and other stress states corresponding to damage strain. The specific data obtained from these studies are presented in Table 5. Notably, the main stress-state parameters of the specimen $\eta \subseteq \left[0.2,-1.1\right]$, the Lode angle parameter $\bar{\theta }=0$, and the plane strains occur in the broad stress state for each PCS specimen.

### 3.3. EBSD Result

To delve into the phase distribution and microstructural attributes of the deformation zone, a scanning electron microscope (SEM) integrated with an electron backscattering diffractometer (EBSD) [38,39] that employed a Carl Zeiss AG (Oberkochen, Germany) Sigma300 system was used. Notably, specimens intended for the analysis of the initial structure were derived from the same heat-treated material as that employed in the compression experiments. To prepare samples for EBSD analysis, vibratory polishing techniques were employed.

**Figure 14:**
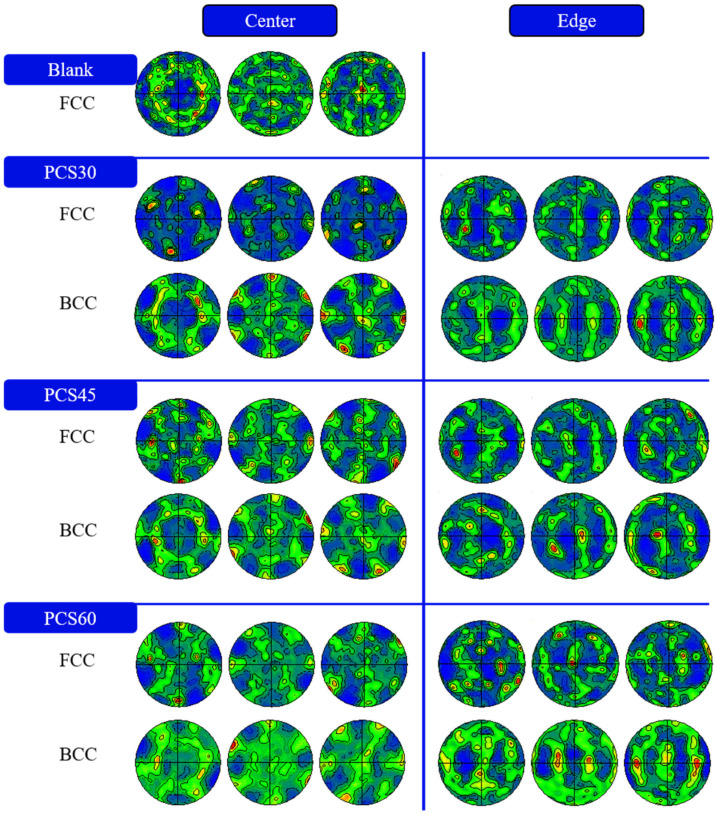
Texture of austenite and martensite at the center and edge of the PCS specimens with various PCAs.

Figure 14 illustrates the polar plot characteristics of PCS specimens at the center and edge positions, featuring PCAs of 30°, 45°, and 60° at a compression of 1.5 mm. Initially, all specimens exhibit FCC austenite phases with strong (100) faceted and (111) filamentary weave features. The notable weakening of the FCC weave strength post-compression is attributed to grain refinement after severe deformation. This is evident in the significant differences in the Lode angle parameter at various locations in the XZ section of the deformed region, as reflected in the weave evolution results.

At the center position, stress triaxiality is the primary consideration and, with an increasing PNA, the hydrostatic pressure and stress triaxiality in the deformation zone decrease, reaching ultra-low stress triaxiality. The FCC exhibits Goss ($(110)\langle 001\rangle$) and copper ($(112)\langle 111\rangle$) weave characteristics with increasing strength. BCC displays similar weave characteristics in the (111) orientation, with the distinction of face-weave features in the (100) orientation.

At the edge position, stress triaxiality decreases with an increasing PNA, while the Lode angular parameter decreases from 0 (representing shear loading) to −1 (representing bi-directional compressive loading). At a lower PNA, the weave characteristics show a weak cube weave, transitioning to an insignificant brass weave with an increasing PNA. The BCC exhibits a weakened plate weave. As the PNA increases, the compression effect strengthens, the strength of the plate weave increases, and significant copper-weave characteristics emerge.

**Figure 15:**
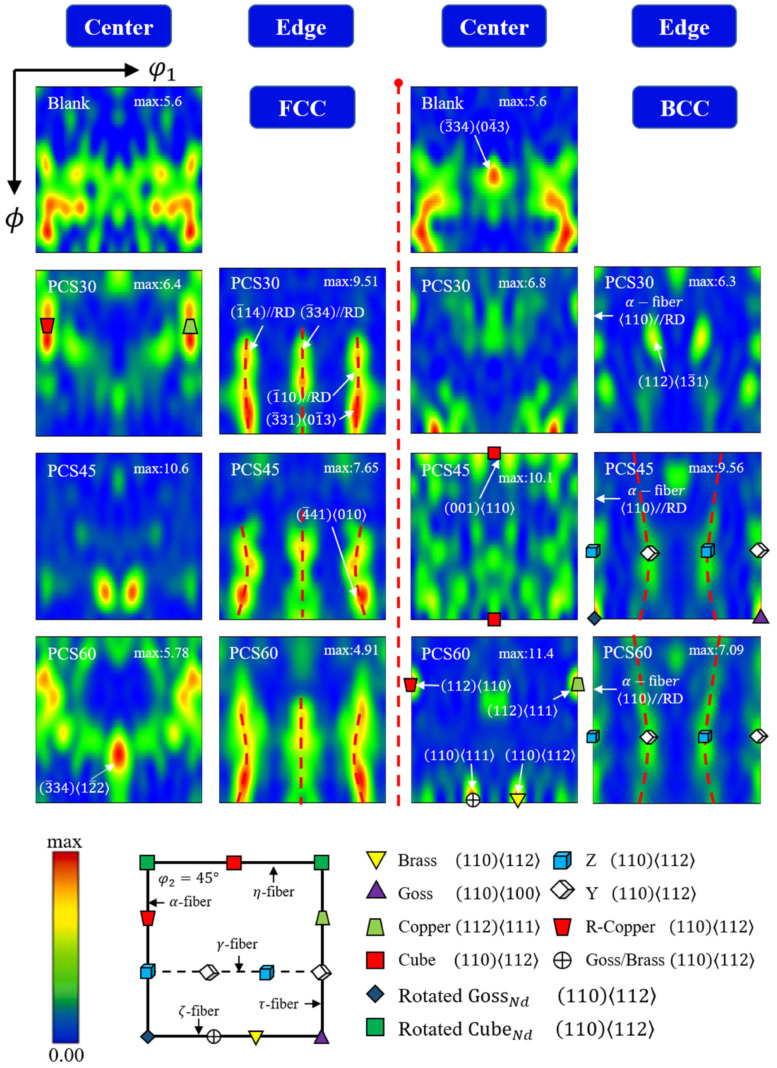
ODF distribution of PCS specimens with sampling points at the center and edge positions and blanks at $\varphi_{2}$ = 45°.

Figure 15 illustrates the results of three-dimensional orientation distribution function (ODF) plots for both the martensite and austenite structures of the PCS specimens at $\varphi_{2}$ = 45° ($\varphi_{2}$ is the coordinate axis in the Euler coordinate system). Regarding the evolution of the FCC structure, at the specimen’s central position, only the effect of stress triaxiality is considered and no significant weaving features are observed. However, at the edge position of the specimen, which is subjected to multidirectional compressive bias loading with the same amount of deformation, noticeable $\left(1\bar{1}0\right)$//RD filament weaving features are present. It is noteworthy that the strength of filament weaving decreases as the preset notch angle (PNA) increases, with the Lode angular parameter tending towards −1.

Concerning the BCC structure of the martensitic phase, there is no evident martensitic selective orientation in the raw material of the specimen. At the center of the specimen, as stress triaxiality decreases, a weave orientation dominated by the cube, brass, and copper types gradually emerges. The strength of the cube weave diminishes with decreasing stress triaxiality, whereas the strength of the other two types of weave increases. At the edge position of the specimen, significant filament weaving features are observed, including α-fiber ((110)//RD), $\alpha^{*}$-fiber ((361)//RD), η-fiber ((100)//ND), and τ-fiber ((110)//TD).

## 4. Discussion

### 4.1. Compression–Shear Effect of PCS Specimens

This study extends the traditional shear sample by enlarging the loaded plane dimensions, ensuring a stable compression loading in the deformation while achieving a $\bar{\theta }=0$ shear loading and a $\bar{\theta }=-1$ planar loading stress state with negative stress triaxiality.

For $\bar{\theta }=0$ pure shear loading, the stress loading characteristic of $\sigma_{2}=\left(\sigma_{1}+\sigma_{3}\right)/2$ exists, typically generated by the tensile groove specimen [33]. In the previous research, the stress triaxiality had been decreased to around 0.1 [25]. However, due to a lack of specimen structures capable of obtaining effective compression stress, the experimental range has not expanded to the η<0 region. PCS specimens successfully address this limitation.

For the $\bar{\theta }=-1$ axisymmetric stress loading state, the stretching and compression testing methods of smooth cylindrical rods and notched cylindrical rods are commonly used [7,40] to cover high $\left(\sigma_{1}>\sigma_{2}=\sigma_{3}>0\right)$, medium $\left(\sigma_{1}=\sigma_{2}>0>\sigma_{3}\right)$, and low ($\sigma_{1}>0>\sigma_{2}=\sigma_{3}$ or $0>\sigma_{1}>\sigma_{2}=\sigma_{3}$) stress triaxiality situations. Similar to Bridgeman’s test method [41], FEM analysis is employed to assess the loading history and stress triaxiality of the specimens, with a methodology akin to PCS specimens. However, existing experiments have not calibrated the FEM and plastic response processes. The deformation process in the loaded plane exhibits a complex stress loading state, requiring additional tests to obtain more data.

Notably, the proposed PCS specimen in this study exhibits planar strain loading characteristics, providing a feasible specimen structure for obtaining a compression–shear combined loading state, facilitating subsequent experimental investigations. When applied, it is essential to consider the uneven stress-state distribution in the deformation, specifically the transition from pure shear to axisymmetric stress states from the center to the edge positions of the XZ cross section of the specimen.

### 4.2. Planar-Shear Stress-State Fracture Process

**Figure 16:**
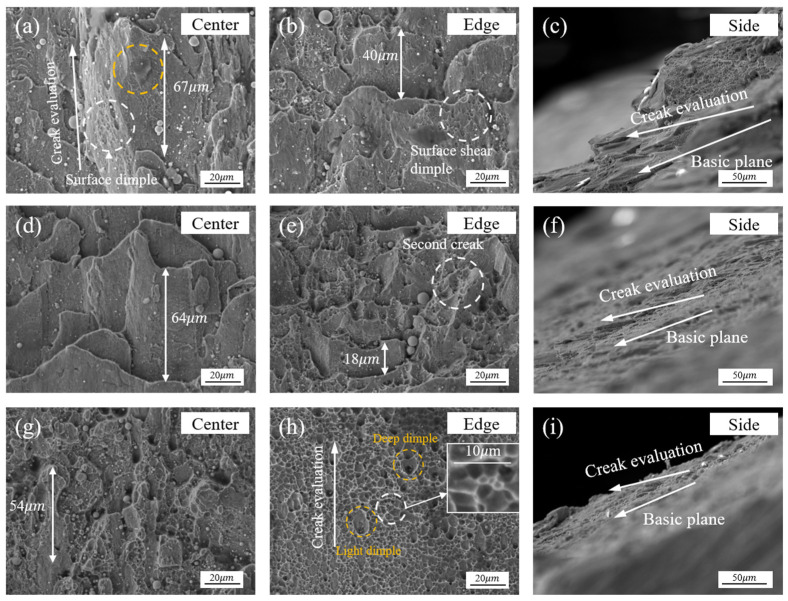
SEM observations at the center, edge, and side of the fractured surfaces of the PCS specimens, showing dimples: (**a**–**c**) PCS30; (**d**–**f**) PCS45; (**g**–**i**) PCS60.

According to the FEM results, the deformation zone in the Z-direction of the specimen exhibits a complex stress character. The bi-directional compression imbalance becomes more intense closer to the edge. This directly affects the fracture process in the Z-direction of the PCS specimens. Specifically, the PCS30 specimen exhibits a notable shear-toughness structure (Figure 16a,b) and a multi-layer, overlapping secondary fracture structure in the direction of fracture height (Figure 16c). The shear ligament surface comprises smaller shear ligament structures, with the ligament size at the center of the PCS45 specimen much larger than that of the edge. In Liu’s [42] study, a mixed fracture structure, consisting of large-sized shear tough nests and secondary fracture tensile tough nests, is also observed. Shear fracture in duplex steels primarily results from martensite cracking, whereas tough nest fracture is a consequence of grain-boundary separation. Isometric tough nests are extensively distributed in the edge region of PCS60 specimens, exhibiting a higher density compared to the central region. These nests demonstrate strong positive stress tensile fracture characteristics.

It can be deduced that the initial fracture position deviates from the central position of the specimen, with the fracture initiating at the edge position before spreading to the central position. The distinction in fracture characteristics between the central and edge locations of the specimens (as shown in Figure 8 and Figure 9) arises from variations in stress states. At the central location, the stress triaxiality is ultra-low and the state is characterized by plane strain, inhibiting the generation and expansion of damage [43]. This limitation suppresses the expansion and evolutionary process of microcracking. Conversely, the edge location experiences higher stress triaxiality compared to the central location, making it more susceptible to damage and fracture. Analyzing the tough-nest width distributions across the three specimens reveals that the central location (whether the shear tough nest or the equiaxial tough nest) has a more significant effect on delayed fracture. Moreover, the tough-nest width at the central location along the deformation direction is notably larger than the width at the edge location.

A transverse fracture was observed at the center position of the PCS30 specimen (Figure 16c,f,i). As the PNA increases, the compression effect becomes more pronounced and the direction of the shear tough fossa fracture is deflected towards the base fracture plane. This deformation trend consistently aligns with that of the base. In summary, as stress triaxiality decreases and the plane-strain effect diminishes, the number of tough inclusions decreases while their size increases. Moreover, with an increase in the degree of shear stress, the fracture mechanism shifts from the equiaxial tough nests caused by tensile stress to shear tough nests. The fracture test results for various stress triaxialities and degrees of shear state indicate a positive correlation between the fracture strain and notch inclination, a negative correlation with the stress triaxiality, and a negative correlation with the absolute value of $\left|\bar{\theta }\right|$.

### 4.3. Effect of Stress State on Microstructure Evolution

Upon analyzing Figure 9 and Figure 10, it can be seen that the PCS specimen deformation zone has different stress states in the center and edge position intervals, and a planar stress state with stress triaxiality less than −1/3 along with bi-directional non-uniform loading is obtained. It is found that, at the center of the specimen, as the stress triaxiality decreases, there is no significant change in the strength of the FCC weave and cube [$\left(001\right)\langle 110\rangle$] and copper $\left[\left(112\right)\langle 110\rangle \right]$ weave structures are gradually formed in the BCC crystals. The weave types along the <110> direction are dominated by cube $\left[\left(001\right)\langle 110\rangle \right]$ and copper $\left[\left(112\right)\langle 110\rangle \right]$ textures. $\left\{hkl\right\}\langle 110\rangle$, as a tensile texture, suggests that during planar compressive loading $\left(\eta <0,\bar{\theta }=0\right)$, grains with different orientations are deflected in the $\langle 110\rangle$ direction. At the same time, the strength of the weave increases and the deformation at fracture increases, showing good ductility [44].

At the edge position of the specimen, which is subjected to bi-directional compressive stress loading, both FCC crystals and BCC crystals exhibit filamentary weaving characteristics, and both are $\left\{hkl\right\}//RD$ oriented. However, as the Lode angular parameter decreases, the FCC weave strength decreases. For BCC crystals, the characteristic orientations of the γ-filament weave, Y-weave, and Z-weave do not increase significantly in intensity and do not form a significant γ-filament weave; instead, they form a non-traditional filament weave characterized by a Y//RD filament weave and a Z//RD filament weave. This reduces the anisotropy of the FCC and BCC crystals, leading to earlier yielding of the materials [45], effectively enhancing the amount of fracture deformation in the RD direction in Figure 16. The increase in specimen plasticity with decreasing stress triaxiality and the increasing bi-directional compressive loading ratio of the specimen is explained in terms of the weave evolution.

## 5. Conclusions

In this study, a compression specimen was constructed with a preset angle between the specimen notch and the compression direction to obtain the multidirectional compressive–shear stress state prevalent in the plastic processing of metals. The stress-state response and plastic processes in the deformation zone were investigated. The main conclusions of the study are as follows:

(1) The compressive deformation of the PCS specimen realizes a deformation zone with the characteristics of compression–shear and bi-directional compression simultaneously. The ratio of bi-direction compressive distribution is affected by the PNA of the specimen.
(2) The center position of the PCS specimen is consistently under compressive–shear loading, and the fracture exhibits a shear dimple. With the transition to the edge position of the specimen and an increase in the PNA, the compression load along the radial direction of the PNA strengthens gradually. This results in a decrease in the Lode angle parameter and the fracture exhibits an equiaxial dimple.
(3) At the central position of the PCS specimen, only the effect of stress triaxiality is considered, and the BCC crystals form significant copper and brass textures as the main plasticizing mechanism. On the other hand, at the edge position of the specimen, which is subject to bi-directional compressive loading, the FCC crystals form a variety of {hkl}//RD-oriented fiber textures. In addition to the α-fiber and τ-fiber configurations, the BCC crystals also exhibit various non-traditional fiber texture features, enhancing plasticity.

## Figures and Tables

**Table 1:** Chemical composition of the 304 stainless steel.

Element	C	Mn	P	S	Si	Cr	Ni	N	Fe
Wt.%	0.08	2	0.035	0.015	0.76	18.5	9.3	0.1	Balance

**Table 2:** The characteristics of stress state in the ductile fracture tests of steel are depicted in Figure 5.

No.	Specimen Type	Analytical Expressions for Stress Triaxiality η	Lode Angle Parameter $\bar{\theta }$
1	Smooth round bars, tension	1/3	1
2	Notched round bars, tension [18]	$1/3+\sqrt{2}ln\;\left(1+a/2R\right)$	1
3	Plastic plane strain, tension	$\sqrt{3}/3$	0
4	Flat grooved plates, tension [33]	$\sqrt{3}/3\left[1+2ln\;\left(1+t/4R\right)\right]$	0
5	Torsion or shear	0	0
6	Cylinders, compression [7]	−1/3	−1
7	Equi-biaxial plane stress, tension	2/3	−1
8	Equi-biaxial plane stress compression	−2/3	1
9	Plastic plane strain, compression	$-\sqrt{3}/3$	0
10	Notched round bars, compression [7]	$-\left[1/3+\sqrt{2}ln\;\left(1+a/2R\right)\right]$	−1
11	Shear plane strain, tension [25]	0.16–0.92	0
12	Shear plane strain, compression (this work)	−0.71–0.16	0

**Table 3:** Stress indices and plastic strains at the center and edge of the fractured sections.

Test No.	Center		Edge	
	$\eta_{ave}$	$\bar{\theta }$	$\eta_{ave}$	$\bar{\theta }$
PCS0	0.143	0	0.091	0.1
PCS30	−0.308	0	−0.212	−0.13
PCS45	−0.686	0	−0.341	−0.35
PCS60	−0.846	−0.15	−0.602	−0.68
PCS90	−0.89	−0.56	−0.593	0

**Table 4:** Strain error between DIC and FEM results.

Test No.	$\varepsilon_{exm}$	$\varepsilon_{FEM}$	RPE	RMSRPE
PCS0	0.24	0.248	3.3%	7.72%
PCS30	0.28	0.301	7.5%	
PCS45	0.39	0.406	4.1%	
PCS60	0.36	0.385	6.9%	
PCS90	0.14	0.158	12.9%	

**Table 5:** Experimental data of the stress states of different structures.

No.	Specimen Type	η	$\bar{\theta }$	$\mathrm{Effective}\;\mathrm{Strain}\;{\bar{\varepsilon }}_{p}$
1	Smooth round bars, tension	1/3	−0.8465	0.59
2	Equi-biaxial tension	2/3	0.9	0.4
3	Shear plane strain [25]	0.92	0	0.247
4	Torsion and shear	0	0	1.1
5	Uni-compression [37]	−1/3	−1	-
6	PCS0	0.143	0	-
7	PCS30	−0.308	0	1.441
8	PCS45	−0.686	0	1.561
9	PCS60	−0.846	0	1.647
10	PCS90	−0.89	0	-

## Data Availability

Data are contained within the article.
